# Natural products: protective effects against ischemia-induced retinal injury

**DOI:** 10.3389/fphar.2023.1149708

**Published:** 2023-04-26

**Authors:** Qianxiong He, Liuyi Xiao, Yuanjiang Shi, Wanrong Li, Xiaorong Xin

**Affiliations:** ^1^ Department of Ophthalmology, Sichuan Provincial People’s Hospital, University of Electronic Science and Technology of China, Chengdu, China; ^2^ Clinical Medicine School of Southwest Medical University, Southwest Medical University, Luzhou, Sichuan, China; ^3^ Department of Ophthalmology, People's Hospital of Golog Tibetan Autonomous Prefecture, Golog, Qinghai, China

**Keywords:** ischemia, neuroprotective, retinal ischemia, natural product, treatment

## Abstract

Ischemic retinal damage, a common condition associated with retinal vascular occlusion, glaucoma, diabetic retinopathy, and other eye diseases, threatens the vision of millions of people worldwide. It triggers excessive inflammation, oxidative stress, apoptosis, and vascular dysfunction, leading to the loss and death of retinal ganglion cells. Unfortunately, minority drugs are available for treating retinal ischemic injury diseases, and their safety are limited. Therefore, there is an urgent need to develop more effective treatments for ischemic retinal damage. Natural compounds have been reported to have antioxidant, anti-inflammatory, and antiapoptotic properties that can be used to treat ischemic retinal damage. In addition, many natural compounds have been shown to exhibit biological functions and pharmacological properties relevant to the treatment of cellular and tissue damage. This article reviews the neuroprotective mechanisms of natural compounds involve treating ischemic retinal injury. These natural compounds may serve as treatments for ischemia-induced retinal diseases.

## Introduction

Maintaining vision health is important in improving the quality of life of the elderly population (Song and Kim, 2021). The World Health Organization reported in the 2019 World Report on Vision that at least 2.2 billion people are either blind or visually impaired, of which at least 1 billion cases of blindness or visual impairment are preventable or have an unclear etiology (Tarik et al., 2019). In addition, the costs associated with eye diseases and visual impairments are unevenly distributed: The burden of responsibility is typically more considerable for older individuals, women, and rural residents in low- and middle-income countries. The Global Burden Study predicted that 834 million people will have some degree of visual impairment by 2050, with this trend increasing over time Study (GBaVICVLEGotGBoD, 2021). Retinal diseases have a significant impact on the preservation of vision, and the development of therapeutic interventions to delay retinal degeneration has been a focus of research in recent decades. Anti-vascular endothelial growth factor (VEGF) drugs are recognized in domestic and international guidelines as the first-line clinical treatment for retinal neovascularization caused by retinal ischemic vascular diseases (Baek et al., 2017). Whereas, long-term, repetitive vitreous cavity injection has side effects, and is inconvenient to administer for long-term treatment (Kim et al., 2019). Additionally, the costs of the treatment are expensive and it remains one of the aspects to take into consideration. Therefore, it is necessary to develop newer, safer, and more effective therapeutic strategies and agents to treat retinal diseases.

Optic nerve is a white-matter tract that contains ganglion cell axons and is also sensitive to the ischemic stimulation. Retinal ganglion cells (RGCs) have been identified as a class of neurons located in the retina and their axons travel through the retinal fiber layer into the optic nerve. RGCs are responsible for transmitting the visual information to the brain through visual pathways (Edo et al., 2020; Ziemba and Simoncelli, 2021). These fibres are essentially axons of the CNS (London et al., 2013). The retinal nerve fiber layer (RNFL) is the innermost layer of the retina and primarily consists of the axons of ganglion cell neurons, which are located below the RNFL (Jonas and Dichtl, 1996). Under ischemic conditions, an insufficient oxygen supply induces injury, apoptosis, and even loss of RGCs, eventually leading to retinal tissue damage (Khalilpour et al., 2017a; Pan et al., 2021). Studies have shown that long-term cerebral ischemic injury can cause retinal damage, affect vision (Xie et al., 2016). Therefore, ischemia-induced neuronal apoptosis has traditionally shown similarities and the presence of proper cellular communication in the blood–retina barrier (BRB) and blood–brain barrier (Madeira et al., 2015; Ramirez et al., 2018).

The clinical entities caused by retinal ischemia including retinal vein occlusion, retinal artery occlusion, diabetic retinopathy, and age-related macular degeneration result in the retinal neovascularization, which is the most common cause contributing to the exacerbation of ischemia-triggered retinal dysfunction (Osborne et al., 2004; Chang and Miller, 2005). Optic nerve diseases originated from chronic or acute ischemic stress such as glaucoma and ischemic optic neuropathy possess the similar pathophysiological disorders including the loss of retinal ganglion cells and their axons, and the damage of optic nerve. The classification of anterior or posterior ischemic optic neuropathy is based on the location of optic nerve damage, as per convention. The most common type of optic neuropathy is anterior ischemic optic neuropathy, which accounts for 90% of cases (Biousse and Newman, 2015; Tournaire-Marques, 2020). Insufficient vascular supply to the optic nerve is the most common cause contributing to the onset of ischemic optic neuropathy, which is featured by acute visual impairment in elder individuals with the age over 50. Research shows that ischemic optic neuropathy is the most common neurodegenerative disorder in older adults, with an estimated incidence of 2.3–10.2 cases per 100,000 people more than 50 years old (Hattenhauer et al., 1997; Preechawat et al., 2007; Tournaire-Marques, 2020). Retina ischemia is characterized by the reduction of retinal vascular flow, the deprivation of nutrients and oxygen as well as energy, and the accumulation of metabolic waste within the retina, which leads to severe lesion in the retina and even the optic nerve (Hattenhauer et al., 1997; Hayreh, 2011). As the extension of central nervous system, retina and optic nerve share the similar physiologic metabolisms as brain such as high oxygen-dependence and high-energy consumption, they therefore are more sensitive and venerable to the ischemic stress. Retinal ischemic injury causes energy-dependent dysfunction, tissue edema, and extensive, irreversible loss of neurons in the ganglion cell layer, which leads to retinal morphological degeneration, functional loss, and eventually, death of ganglion cells (Nagakubo et al., 2019; Lv et al., 2022). In addition, retinal cells are sensitive to ischemia, hypoxia, and energy metabolism. When ischemia persists for 60 min or more, ischemic damage often leads directly to vision loss or blindness, because RGCs are sensitive and fragile cells (Dilsiz et al., 2006; Liu et al., 2019; Qin et al., 2019). However, the extent of retinal tissue damage during ischemia depends on the severity and duration of the circulatory disturbance (Hughes, 1991; Khalilpour et al., 2017b).

Ischemia reduces blood flow, while oxygen depletion depletes substrates, such as oxygen and glucose. Energy depletion causes neuronal depolarization, which leads to the activation of glutamate receptors and increase in intracellular Ca^2+^ concentration (Dirnagl et al., 1999). In addition, the infiltration of proinflammatory elements leads to an increase in free radical generation and reactive oxygen species (ROS) formation, resulting in RGC death through apoptosis (Lo et al., 2003; Juybari et al., 2019).

Various herbal products derived from natural products are grown worldwide, especially in Middle Eastern, European, Asian countries with a history of exploring traditional medicine (Nagakubo et al., 2019; Lv et al., 2022). The therapeutic potential of natural herbal treatments for ischemic retinal damage has become increasingly evident in recent years. These medicines, including *Scutellaria*, lutein, and wolfberry, have antioxidant, anti-inflammatory, and anti-excitotoxic properties and can maintain the permeability of the retinal blood supply (Li et al., 2009; He et al., 2014; Pan et al., 2022). In addition, various natural extracts, such as resveratrol, *Ginkgo biloba* extract, and curcumin, have been shown to protect the CNS and exhibit neuroprotective properties (Yuan et al., 2014; Wang et al., 2017a; Luo et al., 2020). People consume flavonoids in fruits and vegetables daily and experience few side effects (Orhan et al., 2015). A growing number of researchers have found that natural extracts are effective in the prevention and treatment of ischemic diseases and their underlying mechanisms may be similar. These natural products can be classified as neuroprotective agents, that is, antioxidants, excitotoxicity inhibitors, antiapoptotic agents, neuropathic factors, and anti-inflammatory agents (Kang et al., 2010; Majid and Bin, 2011). The neuroprotective and pharmacological properties of these natural extracts provide a solid foundation for various models of ischemic injury. Given their widespread availability, low cost, and low toxicity, natural products may be effective treatments for ischemic diseases and neuropathy. This review will focus on the applications of natural compounds as neuroprotective agents in natural products. Table 1 summarizes the findings for several natural compounds discussed in this paper. (Note: some sequentially numbered references are in the table.)

**TABLE 1:** Preclinical study of natural products in retinal ischemia model.

Compound	Source	Dosage	Time of therapy	Vivo/Vitro	Species	Animal model	Mechanism	Reference
Theissenolactone C	Theisseno cinerea	10 mg/kg	2 days	Vivo,Vitro	Male rat	IOP-I/R	Through activating ERK, NF-κB-dependent pathway	Qi et al. (2013)
Crocin	Saffron	5, 25, 50 mg/kg/day	3 days	Vivo	Male rat	IOP-I/R	By activating PI3K/AKT and ERK pathway	Hashem et al. (2017), Shi et al. (2010)
Pomegranate extract	Pomegranate	250 mg/kg/day	15 days	Vivo	Male rat	IOP-I/R	By activating Nrf2 signalling	Kim et al. (2016)
Caffeic acid phenethyl ester	Propolis	10 µmol/kg/day	7 days	Vivo	Female rat	IOP-I/R	The inhibition of oxidative stress and apoptosis	Otsuka et al. (2016)
Mangiferin	Mango	30 mg/kg/day	7 days	Vivo	Mice	IOP-I/R	Upregulation SIRT1	Lin et al. (2018)
Astaxanthin	Seafood	twice 100 mg/kg/day	4 days	Vivo,Vitro	Mice	ECA, PPA ligated	Against neurodegeneration during ischemic retinopathy	Li et al. (2012), Seong et al. (2017), Li et al. (2020)
		10,100 mg/kg/day	8 days	Vivo	Male rat	Laser-RB	Reduces both RGC death and macrophage infiltration, preserving visual function	
		50 mg/kg	3 days	Vivo	Mice	IOP-I/R	Via the Nrf2/HO-1 pathway	
Haematococcus pluvialis	Astaxanthin	100 mg/kg/day	7 days	Vivo	Male rat	Laser-RB	By mTOR/Akt signaling	Lin et al. (2020)
Resveratrol	Wine and grapes	5,25 mg/kg/day	7 days	Vivo	Mice	IOP-I/R	Up-regulation of eIF2a-CHOP and IRE1a-XBP1 pathways	Majid and Bin (2011), Pang et al. (2020), Zhu et al. (2018), Wu et al. (2020a), Li et al. (2021a), Desai et al. (2022)
		N/A	7 days	Vivo,Vitro	Male rat	IOP-I/R	By increasing Opa1 expression	
		20 mg/kg/day	5 days, 4 weeks	Vivo	Mice	IOP-I/R	Protecting RGC from degeneration, downregulation of caspase-8 and caspase-3 expression	
		250 mg/kg	7 days	Vivo	Male rat	IOP-I/R	Through regulation of the SIRT1-JNK pathway	
		10, 50, 100 μM	7 days	Vivo	Mice	IOP-I/R	SIRT1 activation, through the activation of Akt pathway	
Curcumin	Curcumalonga L.	100 mg/kg	7 days	Vivo	Male rat	Carotid artery ligation	Tthe JNK-mediated apoptosis	Lin et al. (2019)
Vincamine	Apocynaceae Vinca	twice 3.15 mg/kg/day	28 days	Vivo	Male rat	Laser-RB	Maybe PI3K/Akt/eNOS signaling pathway	Huang et al. (2019)
Icariin	Herba Epimedium	100 mg/ml	28 days	Vivo	Male rat	Laser-RB	Via modulation of CEBP-β/G-CSF/noncanonical NF-κB axis	Huang et al. (2020a),Chou et al. (2022), Ren and Zhang (2020)
		5 µM/3µL	N/A	Vivo	Male rat	Laser-RB	Triggered IKKβ to phosphorylate PTEN for AKT activation	
n-Butylidenephthalide	Angelica	10 mg/kg/day	7 days	Vivo	Male rat	Laser-RB	Through inhibition of the NF-κB signaling pathway	Chao et al. (2013)
Houttuynia cordata	Saururaceae	400,1000 mg/kg/day	14 days	Vivo	Male rat	IOP-I/R	Through inhibiting microglia activation	Li et al. (2018a)
Lutein	Spinach, kale	0.2 mg/kg	1 days	Vivo	Mice	MCAO	Decreased NT and nuclear PAR immunoreactivity	Wang et al. (2017)
Baicalein	Scutellaria	10, 100 mM	7 days	Vivo,Vitro	Male rat	IOP-I/R	Downregulation of HIF-1a, VEGF, and MMP-9 and upregulation of HO-1	Yuan et al. (2014), Kim et al, (2020)
		10 mM Ba	28 days	Vivo,Vitro	Mice	IOP-I/R	By PI3K/NF-kB Axis	
Echinacoside	Cistanche salsa	20 mg/kg/day	7 days	Vivo	Male rat	IOP-I/R	Via activation of antioxidant enzymes and suppression of inflammation	Chao et al. (2018)
KIOM-2015E	Aceraceae	100, 200 mg/kg/day	5 days	Vivo	Male rat	MCAO	Inhibit the activation of astrocytes	Arikan et al. (2015)
Dendrobium	Orchidae	0.5 g, 1g/kg/day	7 days	Vivo,Vitro	Male rat	IOP-I/R	By dowregulating placental growth factor and upregulating norrie disease protein	Liu et al. (2013)
Quercetin	Flavonoids	20 mg/kg/day	2 days	Vivo	Male rat	IOP-I/R	By reducing apoptosis	Chao et al. (2020)
Emodin	Frangula bark	0.8 mmol/L	15 days	Vivo	Male rat	IOP-I/R	By CK2 inhibition	Chien et al. (2021), Huang et al. (2018)
		4, 10, 20 μM	2 days	Vivo,Vitro	Male rat	IOP-I/R	Through dowregulating β-catenin and vascular endothelium factor	
Oroxylin A	Scutellariae	15 mg/kg/day	28 days	Vivo	Male rat	Laser-RB	By activating Nrf2 signaling	Pan et al. (2014), Gong et al. (2019)
Sulforaphane	Broccoli	12.5 mg/kg/day	7 days	Vivo	Male rat	IOP-I/R	Through activating Nrf2/HO-1 Pathway	Ambrecht et al. (2014), Wu et al. (2020), Yang et al. (2017)
		5,10,20 mg/kg/day	9 days	Vivo	Female rat	IOP-I/R	Inhibition of the NLRP3 inflammasome activation	
		25 mg/kg/day	5 days	Vivo	Mice	IOP-I/R	Relative changes in electroretinogram (ERG)	
LBP	Lycium barbarum	1 mg/kg/day	7 days	Vivo	Male rat	IOP-I/R	Activation of the Nrf2/HO-1 pathway	Orhan et al. (2015), Huang et al. (2020b), Kara et al. (2014)
		500,250,100 mg/kg	56 days	Vivo	Male rat	IOP-I/R	Antioxidative capacity	
		1, 10 mg/kg	7 days	Vivo	Mice	MCAO	Enhanced immunoreactivity of PKC-α and attenuated GFAP expression	
Algae Oil	Docosahexaenoic acid	1 µL/Kg/day	7 days	Vivo	Male rat	Laser-RB	Inhibits ERK activation	Khalilpour et al. (2018)
Hesperetin	Fruit peel	20 mg/kg	2 days	Vivo	Male rat	IOP-I/R	By inhibiting apoptosis of retinal cells	Kumar et al. (2020)
Rhus coriaria extract	Rhus coriaria	200,400 mg/kg/day	10 days	Vivo	Mice	ONC-Injury	The anti-inflammatory activity	Li et al. (2014)
Allium cepa	A. cepa	300 mg/kg	28 days	Vivo	Mice	ECA, PPA ligated	Increased expression of BCl-2, GDNF, GFAP, and Brn3b	Li et al. (2022)
Tetrandrine	Stephania tetrandra	10 µM/2 µL	1 day	Vivo,Vitro	Mice	IOP-I/R	Activated caspase-3 and Bcl-2	Musayeva et al. (2021)
Gastrodin	Gastrodia elata	10,25,50,100 μM	1 day	Vitro	Precursor cells	OGD/R	Through activating PI3K/AKT/Nrf2 signaling pathway	Rivera-Pérez et al. (2020)
Betulinic Acid	White birch	50 mg/kg/day	8 days	Vivo	Mice	IOP-I/R	Reduction in ROS levels	Yang et al. (2019)
Epigallocatechin 3-Gallate	Green Tea	1.5, 7.5, 15, 30 mg/kg	2 days	Vivo	Rabbit	IOP-I/R	Through activating Nrf2/HO-1 signaling pathway	Zhang et.al. (2008), Romano et al. (1993), Sheng et al. (2019)
		275 mg/kg	14 days	Vivo	Male rat	IOP-I/R	Reduced the increased protein expressions, enhanced the Jak phosphorylation	
		200 ml/day	8 days	Vivo,Vitro	Male rat	IOP-I/R	Attenuates retinal neuronal death	
Glucose	Honghua	1-100 ^mol/lglucose	N/A	Vivo	Male rat	Laser-RB	Intravitreal injection of glucose provided highly significant neuroprotection	Varga et al. (2017)
e 5α-Androst-3β, 5α, 6β-triol	Nephthea brassica	40, 80µg/8µl	N/A	Vivo,Vitro	Male rat, mice	IOP-I/R	By activating Nrf2 pathway	Zhang et al. (2013)
Prunus Cerasus Seed Extract	Sour Cherry	30 mg/kg/day	50 days	Vivo	Male rat	Retinal artery blockage	Through HO-1 dependent mechanism	Davey et al. (2020)
Escin	Aesculus hippocastanum	0.9, 1.8 mg/kg	1 day	Vivo	Male rat	IOP-I/R	May be correlated with the upregulation of occludin	Nguyen et al. (2019)
Zeaxanthin	Marigold	5 μg/μL	7 days	Vivo	Mice	IOP-I/R	Increase in visual acuity, enhances the survival of RGCs	Guan et al. (2020)
Puerarin	Pueraria	10-1000 μM	N/A	Vitro	ARPE19	Hypoxic	Through activating PI3/Akt pathway	Li et al. (2021), Wang et al. (2017)
		25, 50, 100 mg/kg/day	2 days	Vivo	Male rat	IOP-I/R	Through inhibiting the activation of TLR4/NLRP3 inflammasome	
Vitexin	Vitex	25 mg/kg	7 days	Vivo	Male rat	IOP-I/R	By activating Nrf2-related signal pathway	Wang and Ai (2005)
Capsaicin	Chilli	0.01 mg/kg	7 days	Vivo	Mice	IOP-I/R	Anti-inflammatory, through endogenous somatostatin	Nivison-Smith et al. (2014)
Ginkgo biloba extract	Ginkgo	100 mg/kg	19 h	Vivo	Rabbit	IOP-I/R	Reduced b-waves, apoptosis	Kang et al. (2010), Nivison-Smith et al. (2018)
		twice 1, 3, 10 mg/kg/day	3 days	Vivo	Male rat	IOP-I/R	Reduced autophagy	
Vinpocetine	Vinca minor	10–100 μM	N/A	Vitro	Male rat	Hypoxic	Regulates cation channel permeability; NMDA glutamate receptors	Nivison-Smith et al. (2017), Li et al. (2018a), Montezano et al. (2010)

### Neuroprotective effects of natural compounds

**FIGURE 1:**
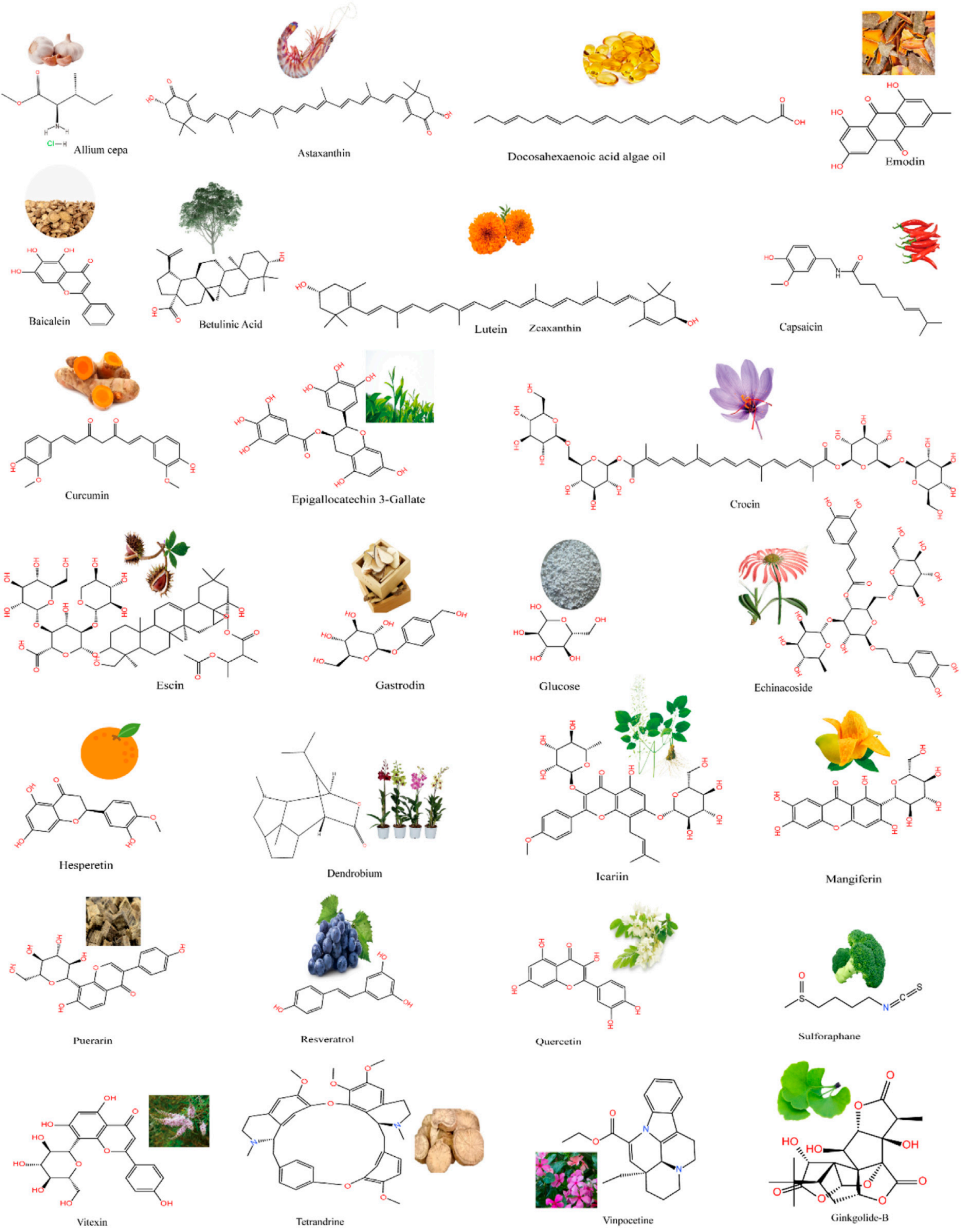
A wide range of natural products from plants and the structural formulae of natural chemical compounds.

**FIGURE 2:**
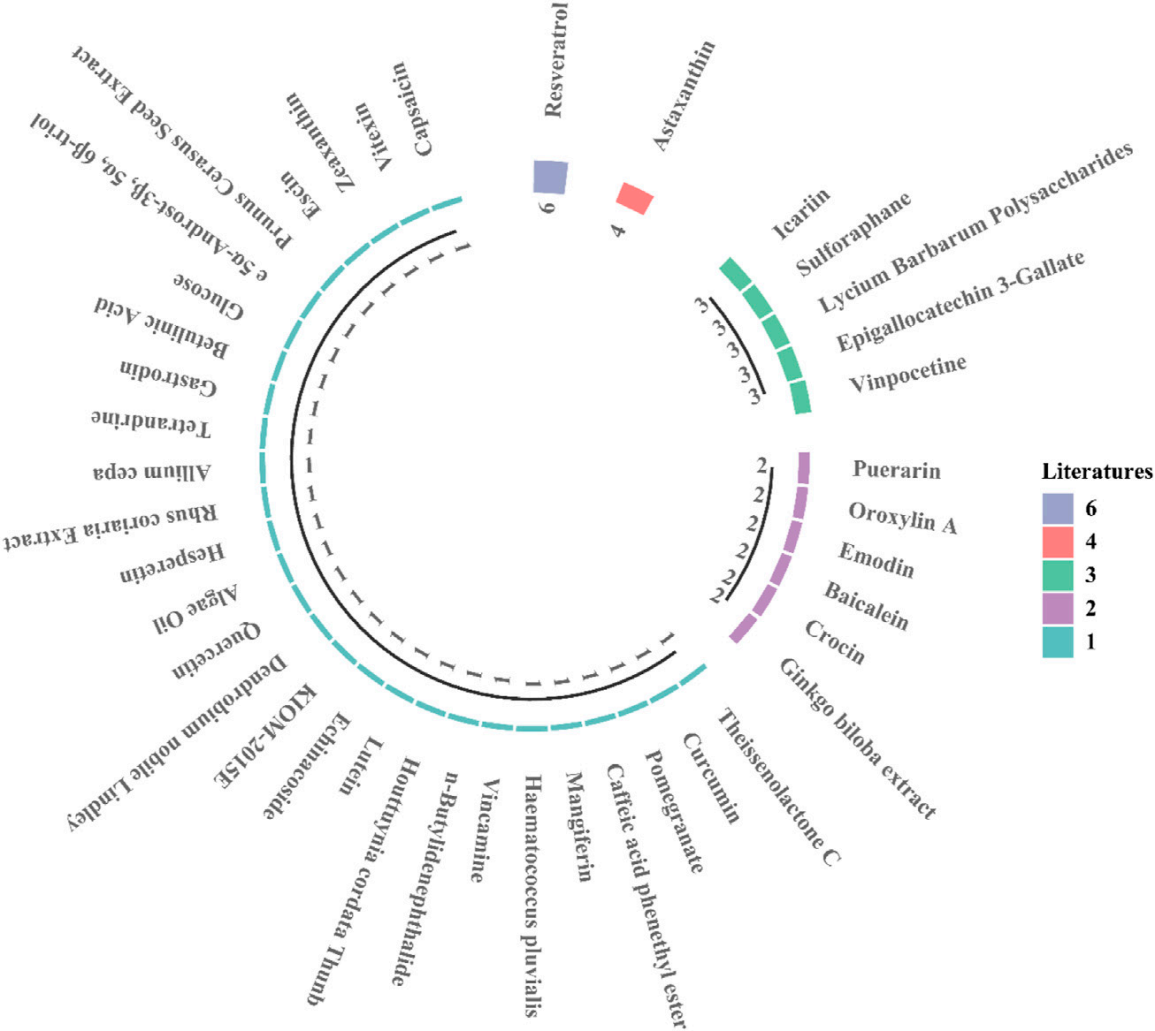
The current number of research articles for each natural compound. Resveratrol: 6 studies; astaxanthin: 4 studies.

Traditional Chinese medicine uses agent with antioxidant, anti-stimulant, anti-neuroinflammatory, antiapoptotic, anti-spasmodic, and anti-aging proportion. Modern pharmacological studies have shown that the active ingredients of natural extracts have preventive effects on neuropathy, inhibitory effects on apoptosis caused by ischemia and hypoxia, beneficial effects on cardiovascular health, and therapeutic effects on neurons in patients with ischemic optic neuropathy. Multiple ophthalmological studies show that conventional drugs can inhibit oxidative stress and inflammatory responses, with prevent apoptosis and RGC loss in retinal ischemia injury (Li et al., 2018a; Huang et al., 2020b; Rivera-Pérez et al., 2020). Natural products (Figure 1) are ideal candidates for treating retinal ischemia. Figure 1 summarizes the molecular structures of natural compounds, such as resveratrol, astaxanthin, icariin, and *Lycium barbarum* polysaccharides (LBPs). These compounds are ranked by the number of studies in which they have been investigated (Figure 2). Taken together, the literature demonstrates the feasibility of developing natural medicines for clinical use.

### Anti-neuroinflammation

The inflammatory response is a regulatory process that prevents excessive damage to the body and represents a pathological provocative state resulting from the dysfunction of body-controlled mechanisms. Inflammation or ischemia can alter the levels of angiogenic factors released by damaged cells (Montezano et al., 2010). The acute inflammatory process begins at the microvascular site near the damaged tissue and induces the infiltration of leukocytes into the surrounding tissue (Woda and Hof, 2011). Research links inflammation to the development of neurodegenerative diseases, such as ischemic optic neuropathy (Guan et al., 2020).

Recent studies have shown that retinal ischemia–reperfusion (I/R) injury leads to an inflammatory response (Li et al., 2018b). Puerarin and sulforaphane have been reported to improve RGC injury caused by retinal I/R by inhibiting the activation of the toll-like receptor 4/nucleotide-binding oligomerization domains, leucine-rich repeats, and pyrin domain-containing 3 (NLRP3) inflammasome (Gong et al., 2019; Guan et al., 2020). The inhibitory activity of theissenolactone-C achieves retinal protection through extracellular regulated protein kinase (ERK)- and nuclear factor-kappa B (NF-κB)-dependent pathways. In addition, it has a strong inhibitory effect on matrix metalloproteinase 9 (MMP-9) activity after I/R injury induced by intraocular pressure (Lin et al., 2019). In another study, the protective mechanism of icariin was characterized by atypical activation of NF-κB induced by the CEBP-β/granulocyte colony-stimulating factor axis, providing long-term neuroprotective effects in patients with optic nerve ischemia through anti-inflammatory effects (Desai et al., 2022). Huang demonstrated that *Epimedium* exerts anti-inflammatory effects in a rodent anterior ischemic optic neuropathy (rAION) model, inhibits nuclear factor-kappa B kinase to phosphorylate phosphatase and alleviate tensin homolog and activate threonine protein kinase (Akt) in response to ischemic stress (Huang et al., 2020a). Further research found that butylens benzonitrile can effectively prevent the apoptosis induced by rAION and that the inhibition of NF-κB signaling can reduce the inflammatory response of blood-derived macrophages infiltrating the optic nerve, thereby alleviating neuroinflammation and facilitating the improvement of RGCs (Chou et al., 2022). The administration of *Houttuynia cordata* after retinal I/R injury has been found to inhibit the upregulation of TNF-α, inducible nitric oxide synthase (iNOS), and IL-1β in the treatment of ischemic events by activating microglia to promote the survival of RGCs (Ren and Zhang, 2020).

*Scutellaria baicalensis* is the main component of many traditional Chinese medicines, and its safety and efficacy in clinical applications have been demonstrated. Baicalin exerts potent anti-inflammatory effects by reducing the induction of proinflammatory cytokines and inhibiting PI3K/NF-κB phosphorylation (Pan et al., 2022). Oroxylin A is a flavonoid that regulates nuclear factor E2-related factor 2 (Nrf2) and its antioxidant enzymes NAD(P)H quinone oxidoreductase 1 and home oxidoreductase 1 (HO-1). Activating these enzymes reduces optic nerve swelling and inflammatory cell infiltration while modulating microglial polarization. Oroxylin A can significantly improve demyelination and optic nerve edema, reduce the number of ED1-positive cells, and effectively relieve ischemia (Huang et al., 2018; Chien et al., 2021), while 5α-Androst-3β-5α-6β-triol can negatively regulate Kelch-like ECH (enoyl-CoA hydratase)-related protein 1 and its downstream blood oxygenase 1 (Sheng et al., 2019). The primary mechanism of *Prunus cerasus* seed extract depends on HO-1 signaling, whereas echinacoside prevents I/R-induced retinal damage by activating antioxidant enzymes and inhibiting inflammation (Varga et al., 2017; Li et al., 2018a). Algae oil inhibits ERK activation to reduce iNOS, IL-1β, TNF-α, and Cl-caspase-3 levels and increase ciliary neurotrophic factor levels in rAION models (Huang et al., 2020b). The expression of caspase-3, caspase-8, superoxide dismutase (SOD) 2, and inflammation-related proteins and the phosphorylation of p38 have been reported to significantly increase in rats with ischemic injury, and green tea extract has been shown to increase the number of surviving RGCs (Yang et al., 2019). Moreover, capsaicin has NF-κB-mediated effects on ischemic injury by inhibiting the anti-inflammatory and retinal protective effects of CXCL10/CXCR3 (Wang et al., 2017b).

### Antioxidant stress

ROS-molecular oxygen (O_2_) reduction metabolites with high biological activities and excessive ROS levels are toxic to cells, eventually causing cell death (Sutherland et al., 2005). Echinacoside administration has been reported to improve retinal morphology, alleviate optic neuritis, and apoptosis, significantly reducing I/R-induced retinal oxidative stress after 7 days (Li et al., 2018a). Crocin is a pharmacologically active ingredient in turmeric that has been reported to significantly increase the level of glutathione (GSH) and SOD and decrease the formation of ROS and the activity of malondialdehyde (MDA) after ischemic retinal injury (Chen et al., 2015). Pomegranate acts as an antioxidant to attenuate retinal structural and functional I/R damage by activating Nrf2 (Hashem et al., 2017). Studies have shown that caffeic caffeic acid phenethyl ester attenuates I/R-induced apoptosis, significantly reduces the level of MDA in rat retinas, and increases the expression of SOD, GSH peroxidase, and catalase in the inner nuclear layer (INL) and RGC layer in rats (Shi et al., 2010).

According to the literature, mangiferin may have neuroprotective properties that could help prevent RGC loss due to oxidative stress. This prevention is achieved by decreasing the levels of hypoxia-inducible factor-1 alpha (HIF-1α) and glial fibrillary acidic protein and increasing the levels of silent mating information regulation 2 homolog 1 (SIRT1) in the retinas of ischemic mice (Kim et al., 2016). In contrast, the anti-RGC oxidation activity of baicalein appears to play a role in the downregulation of MMP-9 (Chao et al., 2013). In *in vitro* and *in vivo* studies, astaxanthin has prevented cell death via concentration-dependent inhibition of ROS, illustrating that astaxanthin inhibits retinal cell death through its antioxidant effects (Otsuka et al., 2016). Endoplasmic reticulum stress plays a crucial role in retinal vascular degeneration by upregulating eukaryotic initiation factor 2 alpha (eIF2α) homologous protein-CHOP and inositol requiring-enzyme 1α-X-box-binding protein 1 pathway, which leads to oxidative stress (Li et al., 2012). Studies have shown that lutein treatment can help reduce cell death and improve outcomes in the eyes subjected to I/R injury because of the ability of lutein to scavenge ROS, reduce inflammation, and protect against cell death signaling pathways. The neuroprotective effects of lutein are associated with reduced oxidative stress (Li et al., 2009).

The protective effects of LBPs and epigallocatechin-3-gallate against I/R injury also involve the activation of the Nrf2/HO-1 retinal antioxidant pathway (He et al., 2014; Rivera-Pérez et al., 2020). Betulinic acid derived from birch can increase the mRNA expression of antioxidant enzymes SOD3 and HO-1 after I/R and reduce the level of ROS (Musayeva et al., 2021). These findings demonstrate the vital role of natural compounds in combating oxidative stress.

### Antiapoptotic process

The apoptotic process is triggered when defective mitochondria cannot be repaired or are removed by the quality control process and ROS reduction mechanisms of cells (Nagata, 2018). This apoptotic process may lead to irreversible loss of function when the retina is damaged (Remé et al., 2000). Many researchers are investigating therapeutic approaches to halting the process of irreversible vision loss by targeting and reducing the apoptotic activity caused by ischemic retinal lesions.

Astaxanthin, a natural product of synthesis of lutein carotenoids biosynthesized by various halogens, can inhibit the apoptosis of RGCs caused by I/R through the Nrf2/HO-1 pathway and relieve the symptoms of severe retinal diseases (Li et al., 2020). The antioxidant effects of vitexin are mediated by the activation of the Nrf2-related signaling pathway in retinal cells and the prevention of RGC apoptosis in RIR rats (Li et al., 2021b). As part of the same retinal I/R model, *G. biloba* extract has been shown to reduce RGC apoptosis (Wang and Ai, 2005; Yuan et al., 2014). Crocin has been observed to prevent rat retinal I/R-induced RGC apoptosis by activating the PI3K/Akt signaling pathway (Qi et al., 2013). A significant reduction in the number of apoptotic cells has been observed in the RGC layer of rat retina after oral administration of astaxanthin. A possible mechanism of the neuroprotective effect is to activate the Akt/mTOR signaling pathway to combat apoptosis and protect against free radical damage (Lin et al., 2018; Lin et al., 2020). Resveratrol acts as a potential neuroprotective agent. Mechanistically, resveratrol treatment significantly downregulates caspase-8 and caspase-3 expression, reduces SOD activity, and inhibits RGC apoptosis. In the mitochondria, resveratrol regulates the ratio of L-Opa1 to S-Opa1, and its protective effect may be mediated through the SIRT1-c-Jun N-terminal kinase (JNK)/SIRT1-Akt pathway (Seong et al., 2017; Wu et al., 2020a; Luo et al., 2020; Pang et al., 2020). Vincamine might protect rats from rAION by affecting the PI3K/Akt/eNOS signaling pathway (Li et al., 2021a). In another study, curcumin inhibited phosphorylated JNK activity in SHR after retinal I/R injury (Wang et al., 2017a). Baicalein prevents retinal ischemia by exerting antiapoptotic effects, upregulating HO-1, and downregulating HIF-1α and VEGF (Chao et al., 2013). Research has shown that leaf extract KIOM-2015E prevents RGC degeneration in posterior rats following MCAO-induced (I/R) models (Kim et al., 2020). Quercetin has been found to exhibit protective effects against I/R injury in the retina by inhibiting the apoptosis of cells in the INL, suggesting that other natural flavonoids, such as hesperetin and naringenin, may have similar protective effects (Kara et al., 2014; Arikan et al., 2015). Primary cultured RGCs treated with tetrandrine maintain the mitochondrial membrane potential and inhibit caspase-3 and Bcl-2 expression after I/R damage (Li et al., 2014). In retinal precursor cells (R28), gastrodin induces the PI3K, Akt, and Nrf2 signaling pathways protectively against oxygen and glucose deprivation/reoxygenation-induced injury (Li et al., 2022).

When administered orally, one dose of epigallocatechin gallate decreases retinal neuronal death and the apoptotic response to light *in vitro*. Puerarin and epigallocatechin gallate similarly protect against hypoxia-induced apoptosis in human retinal pigment epithelial cells (ARPE19) by activating the PI3K/Akt pathway (Zhang et al., 2008; Nguyen et al., 2019). These findings provide support for further investigation of natural compounds as novel protective agents against retinal ischemia.

### Anti-excitotoxicity

During ischemic injury, the pathophysiological process of neuronal damage involves two excitatory amino acid transmitters: glutamate and aspartate. In excess, these neurotransmitters cause excitotoxicity, resulting in neuronal degeneration and a marked increase in extracellular concentrations of glutamate (Rothman and Olney, 1986; Choi and Rothman, 1990). The neuroprotective effect of the Chinese herbal medicine Honghua extract involves the inhibition of excitotoxicity (Romano et al., 1993). As another critical discovery, vinpocetine protects inner retinal neurons with functional NMDA glutamate receptors against retinal ischemia (Nivison-Smith et al., 2018).

### Other effects

Previous research has demonstrated that the administration of sulforaphane dramatically reduces the loss of retinal function caused by ischemia and induces pronounced thickening of the inner retinal layer (Ambrecht et al., 2014). Daily administration of LBP has been shown to effectively alleviate ischemia-induced retinal dysfunction and reduce associated neuronal death and glial cell activation (Yang et al., 2017). In mice injured by optic nerve crush, treatment with ethanolic extract of *Rhus coriaria* and linoleic acid yields anti-inflammatory effects (Khalilpour et al., 2018). Furthermore, research has shown that vinpocetine reduces intracellular cation channel permeability and apoptosis (Nivison-Smith et al., 2014; Nivison-Smith et al., 2017). In addition, emodin may protect neurons from ischemic retinal injury by downregulating β-catenin/VEGF protein expression during ischemia, while *Allium cepa* pretreatment protects I/R mice from retinal neuronal damage by regulating neurotrophic factors (Chao et al., 2020; Kumar et al., 2020). Interestingly, escin has been observed to have a synergistic protective effect against BRB disruption (Zhang et al., 2013). Finally, *Dendrobium nobile* Lindley can prevent retinal ischemic/hypoxic changes by downregulating the level of placental growth factor and upregulating the level of Norrie disease protein (Chao et al., 2018).

### Clinical application

The significance of herbal medicine cannot be overstated in the betterment of patients afflicted with diverse illnesses. With supplementation of diet with herbal substances containing phytochemicals, the efficacy of conventional medication has been observed to increase. In the clinical treatment of retinal diseases, the primary means of assessing treatment outcomes include the evaluation of the best-corrected visual acuity (BCVA), central macular thickness (CMT), blood flow, macular pigment optical density (MPOD), and contrast sensitivity; optical coherence tomography; angiography; and electroretinogram (ERG).

Recently, phytochemicals have been shown to have the potential for beneficial effects on retinal diseases (Wimpissinger et al., 2007; Li et al., 2021c). A placebo-controlled randomized clinical trial evaluated the BCVA and CMT of patients before and after the intervention and every month thereafter for 3 months. It demonstrated a noteworthy distinction between log MAR changes in the 15-mg-crocin and placebo groups before and after the trial. Daily consumption of 15 mg of crocin proved effective in treating diabetic macular edema (DME), significantly decreasing the macular thickness and improving the BCVA (Yorgun, 2019). In another research, the oral administration of saffron significantly increased the amplitude of the fERGs of patients when compared with both baseline and placebo supplementation. In addition, the fERG thresholds were observed to decrease after saffron supplementation but not after placebo supplementation in comparison with the baseline values. These data indicate that consuming saffron supplements may lead to rapid and significant improvements in retinal function in individuals with early AMD (Falsini et al., 2010).

A study of patients undergoing curcumin therapy for 3 months showed that 84% of patients experienced an improvement in their visual acuity, and 92% exhibited a decrease in macular edema. These findings suggest that curcumin–phospholipid could be a beneficial treatment option for diabetic retinopathy (Mazzolani et al., 2018). Several clinical trials have shown that daily consumption of *L. barbarum L.* as a dietary supplement can help preserve the visual acuity and macular structure and increase the MPOD (Bucheli et al., 2011; Chan et al., 2019; Li et al., 2021c).

Clinical trials have revealed that several natural herbal compounds can dilate the blood vessels and augment blood flow. A randomized, double-blind, placebo-controlled study showed a significant increase in the macular square blur rate and choroidal blood flow velocity after 4 weeks of AXT ingestion compared with pre-ingestion values (Saito et al., 2012). Puerarin exhibits properties that can lower blood viscosity, enhance microcirculation, and effectively treat diabetic retinopathy (Ren et al., 2000). Oral consumption of antioxidants (i.e., *G. biloba*) for a month has been shown to increase ocular blood flow in the retinal and retrobulbar vascular beds in individuals with glaucoma (Wimpissinger et al., 2007; Harris et al., 2018).

Currently, the clinical management of ischemic retinal injury-related diseases involves the utilization of *G. biloba* leaves and compound lauryl injections. A clinical phase 3 trial (NCT02388984) among 480 participants is currently underway to assess the effectiveness and safety of Danshen dripping pills in treating diabetic retinopathy. The trial is double-blind and randomized. Meanwhile, another clinical trial (NCT04117022) is recruiting participants to evaluate the capacity of ERG to identify changes in global retinal function after treatment with carotenoid vitamin supplements in patients with diabetic retinopathy.

### Limitations

The primary limitation of clinical trials is the restricted sample size. This is attributed to various factors, such as differences in the treatment response and age of patients, execution criteria, and feedback authenticity. Additionally, discrepancies may arise in the judgment of investigators, inadequate follow-up time, and lack of placebo control. Furthermore, uncontrollable factors such as environmental and spatial factors may also affect the study outcomes. Therefore, it is crucial to consider all aspects of study design thoughtfully.

### Toxic and side effects of natural products

Although natural products are widely used for disease prevention and treatment, limited information is available regarding their mechanism of action and potential toxicity. The most frequently observed toxic side effect of natural products is hepatorenal toxicity, which can result in metabolic disturbances, electrolyte imbalances, acute kidney injury, chronic kidney disease, and even mortality.

Most present-day mechanistic exploratory investigations have been conducted to assess the toxicology, safety, and efficacy of the active components of natural products. Mechanistic studies or clinical trials of potent pharmacological active ingredients are frequently conducted within the safe dosage limits. Natural products generally exhibit a milder toxicity profile than do synthetic drugs. Clinical trials have revealed minimal adverse reactions, although one such trial has reported increased appetite, swollen feet, and stomach pain among patients who were administered saffron without any other notable adverse effects (Yorgun, 2019).

## Discussion

Most neurodegenerative diseases are accompanied by apoptosis, stress, and inflammatory response; ocular involvement is common and often leads to decreased vision or vision loss (McKee et al., 2011; Cordeiro et al., 2017). Many proteins are involved in cellular injury following ischemia and hypoxia, including HIF-1α, SIRT1, Nrf2, NF-κB, ERK, p38, PKC, and NLRP3 (Sun et al., 2001; Mishra et al., 2018; Hong et al., 2020; Parker et al., 2020; Song et al., 2020). The literature demonstrates that neuroprotective therapy is crucial from a strategic perspective.

Ischemic retinal injury is caused by several factors, including diabetic retinopathy, DME, glaucoma, AMD, ischemic optic neuropathy, and retinal I/R injury. Neurons undergo apoptosis when they do not receive adequate signals (Claes et al., 2019). Inflammatory cytokines, including TNFα and FasL, which is membrane-bound, can directly trigger apoptosis (Gregory et al., 2011; Roh et al., 2012). Low-grade inflammation leads to a sequence of cellular abnormalities and injury to the retina tissue because of the increased presence and significant involvement of proinflammatory mediators, adhesion molecules, chemokines, and growth factors in the development of ischemic retinal injury (Tang et al., 2023). When injury or stress occurs in the retina, inflammatory cells are activated, and harmful stimuli trigger endothelial cells and pericytes to secrete proinflammatory factors. In response to proinflammatory cytokine stimulation, endothelial cells produce intercellular adhesion molecules, attracting leukocyte adhesion to capillaries. Once attached, these leukocytes cause capillary blockage and disrupt the tight junctions between endothelial cells (Lutty, 2013). *In vitro* findings suggest that inflammatory cytokines may be the primary factor responsible for apoptosis (Doganay et al., 2002).

Oxidative stress has been studied as part of the pathogenesis of complex retinal diseases. During apoptosis, mitochondrial ROS cause oxidative stress, which has a role in apoptosis. Oxidative stress-inducing ROS are naturally produced through aerobic metabolism and can also be generated by external factors, such as ultraviolet light, smoke, and heavy metals (Fletcher, 2010). Fortunately, eukaryotic cells have established protective strategies to combat ROS by enhancing endogenous antioxidant production (Lo et al., 2006). Hypoxia, free radicals, and toxins can produce ROS and damage the cell membrane, resulting in DNA damage and apoptosis (Auguy et al., 2015). The primary source of ROS is mitochondrial electron transport chain complex I, which produces superoxide anion (O_2_^−^) (Sinha et al., 2015), which can be further oxidized by NOS, generating peroxynitrite (ONOO−). The lipid peroxidation products formed by peroxynitrite are 4-hydroxynonenal and MDA, which have been shown to induce neuronal apoptosis (Aoyama et al., 2012). Notably, researchers have discovered that flavonoids can prevent vision loss caused by ischemic retinal damage through apoptosis (Dua et al., 2015). This process involves multiple cell types, including neurons and glial cells. During this process, activated microglia produce several inflammatory mediators that stimulate inflammation, such as cytokines and chemokines. Many factors can trigger microglial activation, including physical injury, neurodegeneration, infection, or neuroinflammatory diseases, such as ischemic optic neuropathy (Airaksinen et al., 2008; Al Mamun et al., 2020). Microglia release inflammatory mediators associated with neurodegeneration, and the CNS expresses proinflammatory mediators. The IL-1β level is significantly increased in the optic nerve head tissue in patients with ischemic optic neuropathy (La Morgia et al., 2016). This increase in the IL-1β level may be related to the loss of RGCs or axons due to degenerative changes. Natural extracts have been shown to ameliorate inflammation-induced necrosis of RGCs, preventing the worsening of optic nerve degeneration (Wang et al., 2017a). In addition, glutamate, a neuroexcitotoxic substance, plays a significant role in the death of RGCs, resulting in reduced or impaired vision (Khalilpour et al., 2017b). Furthermore, studies have shown that natural products minimize the damage caused by excitotoxicity through their neuroprotective properties (Yang et al., 2019).

## Conclusion

Ischemic retinal injury is the fundamental pathological mechanism for various ophthalmic ailments, including ischemic optic neuropathy, AMD, and I/R injury. Notably, although the findings of some clinical trials conducted with herbs have promising, they offer strategies for the clinical treatment of ischemic retinal diseases, with broad implications for reversing the visual function. Therefore, each strategy strives toward achieving a deeper comprehension of the etiology of retinal diseases and the evolution of potential treatments in their respective fields. This will help clinicians better understand the efficacy and full potential of herbs.

**FIGURE 3:**
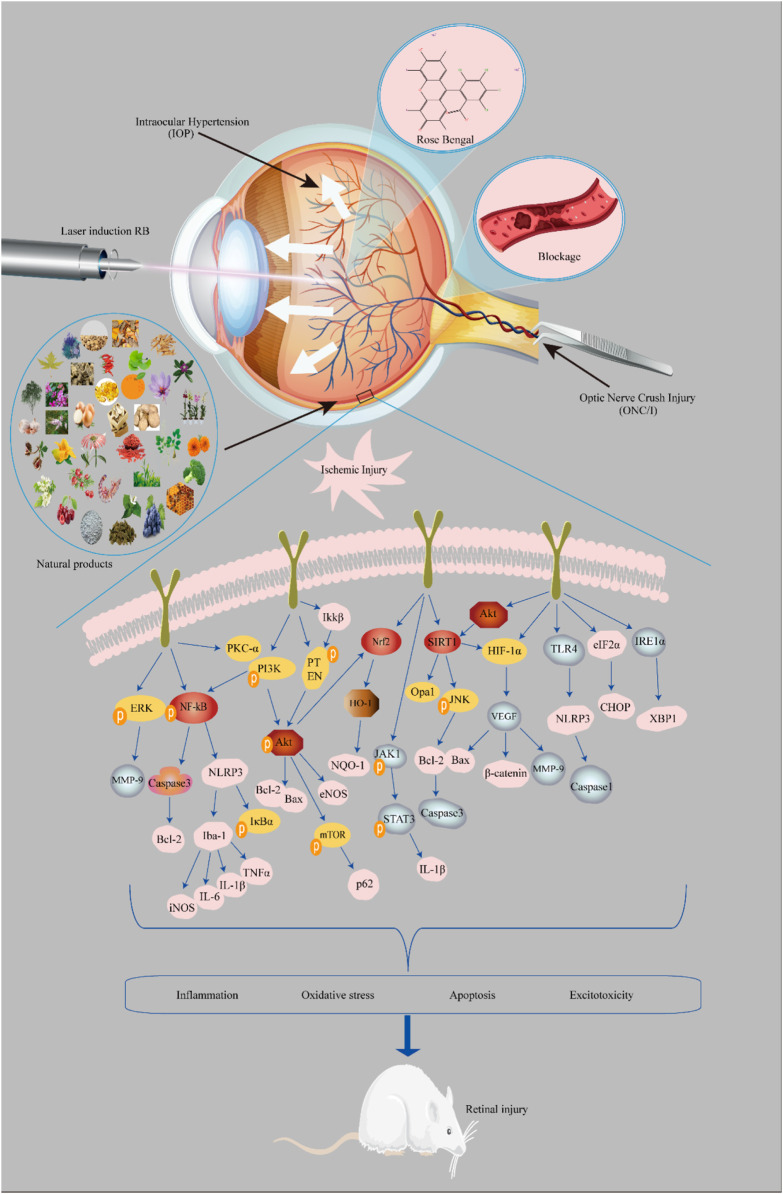
Mechanisms of the neuroprotective effects of natural compounds involved in retinal ischemic injury. Model: 1) intraocular hypertension, 2) laser induction with rose bengal, 3) vascular occlusion, and 4) optic nerve crush injury.

This review summarizes the mechanisms of ischemic retinal injury and the potential role of neuroprotective agents (Figure 3). Neuroprotection involves the slowing or blocking of processes leading to nerve cell death in neurodegenerative diseases. Natural compounds have the advantages of being widely available and inexpensive and having few side effects. In summary, considering the significant potential of natural compounds in treating retinal ischemic injury diseases, it is necessary to further develop and conduct in-depth research.
